# Effects of a Soluble Guanylate Cyclase Stimulator Riociguat on Contractility of Isolated Pulmonary Artery and Hemodynamics of U46619-Induced Pulmonary Hypertension in Dogs

**DOI:** 10.3390/vetsci10020159

**Published:** 2023-02-15

**Authors:** Satoshi Kameshima, Yuki Nakamura, Kenji Uehara, Tomoko Kodama, Hideyuki Yamawaki, Kotaro Nishi, Shozo Okano, Ryo Niijima, Yuya Kimura, Naoyuki Itoh

**Affiliations:** 1 Laboratory of Small Animal Internal Medicine 1, School of Veterinary Medicine, Kitasato University, Higashi 23 Bancho 35-1, Towada 034-8628, Aomori, Japan; 2 Laboratory of Veterinary Pharmacology, School of Veterinary Medicine, Kitasato University, Higashi 23 Bancho 35-1, Towada 034-8628, Aomori, Japan; 3 Laboratory of Small Animal Surgery 2, School of Veterinary Medicine, Kitasato University, Higashi 23 Bancho 35-1, Towada 034-8628, Aomori, Japan; 4 Small Animal Teaching Hospital, School of Veterinary Medicine, Kitasato University, Higashi 23 Bancho 35-1, Towada 034-8628, Aomori, Japan

**Keywords:** cardiac output, cyclic guanosine monophosphate, dog, endothelial dysfunction, pulmonary arterial contractility, pulmonary hypertension, pulmonary vascular resistance, riociguat, soluble guanylate cyclase

## Abstract

**Simple Summary:**

Pulmonary hypertension (PH) is a progressive and refractory vascular disease in both dogs and humans. The soluble guanylate cyclase stimulator riociguat is a relatively novel pulmonary vasodilator used for the treatment of PH in human medicine. Although riociguat may have beneficial effects in the treatment of PH in dogs, there is a lack of basic studies on its clinical application in veterinary medicine. Thus, the aim of this study was to examine the effects of riociguat on the contractility of an isolated canine pulmonary artery (PA) and the hemodynamics of acute PH model dogs. In an isolated canine PA, riociguat endothelium independently increased cyclic guanosine-3′,5′-monophosphate (cGMP) levels and reduced vasocontractile endothelin-1-induced contraction. Moreover, riociguat inhibited the increased pulmonary vascular resistance and elevated PA pressure in thromboxane A2 analog U46619-induced PH model dogs. In contrast, riociguat had no effect on basal systemic arterial pressure. These results suggest that riociguat can inhibit the elevation of PA pressure through PA relaxation via an endothelium-independent increase in cGMP levels in dogs with PH. Riociguat may be a novel and safe therapeutic agent for the treatment of PH in veterinary medicine.

**Abstract:**

Soluble guanylate cyclase (sGC) stimulator riociguat is a relatively novel therapeutic agent for pulmonary hypertension (PH) in human medicine. Riociguat induces endothelium-independent pulmonary artery (PA) relaxation by directly activating the sGC-cyclic guanosine-3′,5′-monophosphate (cGMP) pathway in muscle cells. Although riociguat may be effective in the treatment of dogs with refractory PH, basic studies on its clinical application in veterinary medicine are lacking. The present study aimed to explore the effects of riociguat on the contractility of an isolated canine PA and the hemodynamics of dogs with acute PH. In an isolated endothelium-denuded canine PA, the effects of riociguat on endothelin (ET)-1-induced contraction and cGMP levels were investigated using the Magnus method and ELISA, respectively. The effect of riociguat on the hemodynamics of the thromboxane A2 analog U46619-induced PH model dog was examined by invasive catheterization. Riociguat increased cGMP levels and reduced ET-1-induced contraction of the isolated PA. Riociguat inhibited the U46619-induced elevation of PA pressure and pulmonary vascular resistance and increased cardiac output, but it had no effect on basal systemic blood pressure. These results demonstrate for the first time that riociguat can inhibit the elevation of PA pressure through PA relaxation via an endothelium-independent increase in cGMP in dogs with PH.

## 1. Introduction

Pulmonary hypertension (PH) is a progressive and refractory vascular disease characterized by a persistent increase in the pulmonary artery (PA) pressure (PAP). PAP elevation is caused by abnormal hemodynamic states, including increased pulmonary vascular resistance (PVR), increased blood flow, and elevated PA wedge pressure (PAWP) [1,2,3].

PH in dogs is divided into six groups: pulmonary arterial hypertension, PH due to left heart disease, PH secondary to respiratory disease or hypoxia, pulmonary vascular embolism, parasitic disease-related PH, and PH with multifactorial or unclear mechanisms [4]. PA abnormalities, including increased contraction, medial and/or intimal wall hypertrophy, collagen accumulation, endothelial dysfunction, and microthromboembolism can promote PH development, which leads to right-sided heart failure and, eventually, death [5,6,7].

The nitric oxide (NO)-cyclic guanosine-3′,5′-monophosphate (cGMP) pathway mediates PA relaxation [8]. Soluble guanylate cyclase (sGC) activated by endothelium-derived NO promotes the conversion of guanosine-5′-triphosphate to cGMP, which leads to vascular relaxation via activation of protein kinase G and a subsequent decrease in intracellular calcium concentration [9]. The phosphodiesterase (PDE) 5 inhibitor, a pulmonary vasodilator, is mainly used to suppress the development of PH in dogs [4]. Under PH conditions, PDE5 inhibitors can reduce PA contraction by inhibiting PDE5, which converts cGMP into the inactive form 5′-GMP [10,11,12]. However, the vasorelaxant effect of PDE5 inhibitors can be attenuated by a decrease in the endothelial NO-dependent conversion of cGMP caused by endothelial dysfunction [13,14]. As a matter of fact, it has been reported that dogs with PH have low reactivity to the administration of the PDE5 inhibitor sildenafil [15,16].

In human medicine, the sGC stimulator riociguat has been approved as a novel therapeutic agent for pulmonary arterial hypertension and chronic thromboembolic PH. sGC stimulators can induce the NO-independent activation of sGC [17], which leads to pulmonary vasorelaxation even in the presence of endothelial dysfunction [18]. Thus, it is possible that riociguat may have efficacy on refractory PH with resistance to PDE5 inhibitors in dogs. Since there is no basic study aiming to make riociguat a therapeutic agent for PH in the field of veterinary clinical medicine, we examined the effects of riociguat on the contractility of isolated canine PA and the hemodynamics of acute PH model dogs in this study.

## 2. Materials and Methods

### 2.1. Animals

Three male beagle dogs [median (range); 11.0 (9.4–11.3) kg body weight] were used to measure the contractility and cGMP concentration of isolated PA. Three male and two female beagle dogs [11.0 (9.3–13.0) kg] were used for hemodynamic evaluation under anesthesia. We confirmed that all dogs were clinically normal based on the results of the physical examination, echocardiography, chest radiography, and blood tests. The dogs were housed individually in cages under a 12/12 h light/dark cycle with constant temperature and humidity.

### 2.2. Drugs

Riociguat (MedChemExpress, South Brunswick, NJ, USA), ET-1 (Peptide Institute, Osaka, Japan), and U46619 (9,11-dideoxy-9α,11α-methanoepoxy prostaglandin F2α; Cayman Chemical, Ann Arbor, MI, USA) were used. In the in vitro study, riociguat and ET-1 were dissolved in dimethyl sulfoxide (DMSO) and sterile water, respectively. For the in vivo study, riociguat and U46619 were diluted with sterile saline.

### 2.3. Measurement of Isometric Contraction of Isolated PA

After deep anesthesia with pentobarbital (100 mg/kg, IV), dogs were euthanized by intravenous injection of potassium chloride solution (1 mEq/mL). The intrapulmonary arteries isolated from the accessory lobe of the lung were dissected into rings (length, 2–3 mm; diameter, 1 mm). The intima was removed from the isolated PA by rubbing with forceps, and intimal removal was confirmed by the elimination of acetylcholine (10 mM)-induced relaxation. Isometric contractions were measured in Krebs solution containing the following (in mM): NaCl, 119.0; KCl, 4.8; KH_2_PO_4_, 1.2; MgSO_4_, 1.2; CaCl_2_, 2.5; NaHCO_3_, 24.9; glucose, 10.0. The absolute contraction was measured in a high-K^+^ solution containing the following (in mM): NaCl, 70.2; KCl, 72.7; CaCl_2_, 1.5; NaHCO_3_, 23.8; MgCl_2_, 1.0; glucose, 5.6; ethylenediaminetetraacetic acid, 0.01. These solutions were saturated with a 95% O_2_–5% CO_2_ mixture at 37 °C and pH 7.4. PA contractility was isometrically measured and digitally recorded using a force displacement transducer (Nihon Kohden, Tokyo, Japan) and PowerLab system (ADInstruments, Colorado Springs, CO, USA). Each PA ring was attached to a stainless steel needle in a 3 mL organ bath under a resting tension of 0.3 g. After equilibration for 30 min, the PA rings were repeatedly exposed to the high-K^+^ solution until the responses stabilized. Concentration–response curves for ET-1 (0.1–100 nM) were obtained by a cumulative application after pretreatment with riociguat (100 nM) or DMSO (control) for 15 min. The pD_2_ value (negative log of EC_50_ of agonist) was calculated using the GraphPad Prism software (GraphPad Software version 8.4.3, San Diego, CA, USA).

### 2.4. Measurement of cGMP Contents

After endothelium-denuded PA was soaked in Krebs solution for 30 min under resting tension (0.3 g), 100 nM riociguat was added for 15 min. Proteins were extracted and stored at −80 °C until subsequent examination. The protein concentration was determined using the bicinchoninic acid method (Thermo Scientific, Rockford, IL, USA). The cGMP concentration of the protein was measured using a cGMP ELISA kit (Enzo Life Sciences, Farmingdale, NY, USA), following the manufacturer’s instructions.

### 2.5. Anesthesia

After premedication with midazolam (0.2 mg/kg; IV), buprenorphine (0.02 mg/kg; IV), and atropine (0.025 mg/kg, SC), the dogs were anesthetized with propofol (6 mg/kg; IV) and intubated. Anesthesia was maintained by the inhalation of 2% isoflurane with 100% oxygen. The end-tidal partial pressure of carbon dioxide (EtCO_2_) and blood oxygen saturation (SpO_2_) were measured and maintained at approximately 40 mmHg and 96–100%, respectively. Heart rate, EtCO_2_, and SpO_2_ were monitored using a biological information monitor (BioScope AM130, Fukuda ME, Tokyo, Japan). Respiration was controlled using a ventilator (KVS-2100, Kohken Medical, Tokyo, Japan), and respiratory rate, tidal volume, and inspiratory time were maintained at 10 breaths/min, 20 mL/kg, and 1.4 s, respectively. The fluid loss was replaced with lactated Ringer’s solution. After the examination, the dogs were allowed to recover from anesthesia.

### 2.6. Invasive Hemodynamic Evaluation

The anesthetized dogs were positioned in right-lateral recumbency. A 4-Fr saline-filled catheter was inserted into the left carotid artery to measure systemic arterial pressure (SAP) using a transducer (Edwards Lifesciences, Irvine, CA, USA) and a biological information monitor (BP-608 Evolution, Omron Healthcare, Kyoto, Japan). A saline-filled Swan–Ganz catheter (5-Fr thermodilution catheter 132F5, Edwards Lifesciences) was inserted through the left jugular vein and advanced into the main PA. The Swan–Ganz catheter was connected to a PowerLab system (AD Instruments), and PAP, central venous pressure (CVP), PAWP, and cardiac output (CO) were measured. The CO was calculated using a thermodilution method based on previous reports [19,20]. The average of the three measurements is presented as the result.

The PVR and systemic vascular resistance (SVR) indices were calculated using the following formulas [21,22]:

$$\mathrm{Body}\;\mathrm{surface}\;\mathrm{area}\;\left(\mathrm{BSA};{\;m}^{2}\right)=\frac{10.1\times \mathrm{body}\;\mathrm{weight}\;{\left(g\right)}^{\frac{2}{3}}}{10^{4}}$$

$$\mathrm{PVR}\;\mathrm{index}\;\left(\mathrm{dynes}\cdot s\cdot {\mathrm{cm}}^{-5}\cdot m^{2}\right)=\frac{\left(\mathrm{mean}\;\mathrm{PAP}\;-\;\mathrm{mean}\;\mathrm{PAWP}\right)}{\mathrm{CO}}\times 80\times \mathrm{BSA}$$

$$\mathrm{SVR}\;\mathrm{index}\;\left(\mathrm{dynes}\cdot s\cdot {\mathrm{cm}}^{-5}\cdot m^{2}\right)=\frac{\left(\mathrm{mean}\;\mathrm{SAP}\;-\;\mathrm{mean}\;\mathrm{CVP}\right)}{\mathrm{CO}}\times 80\times \mathrm{BSA}$$

### 2.7. Study Protocol

**Figure 1:**
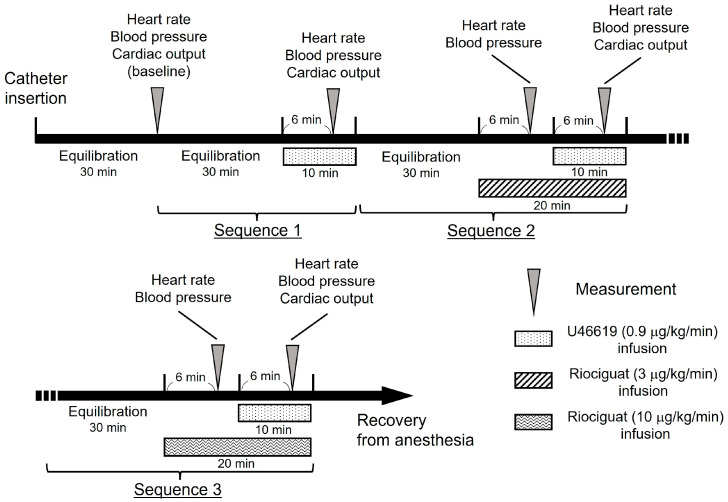
Timeline of the study protocol. The drug sequences 1 to 3 were randomized for each dog.

After catheter insertion, blood pressure and CO were measured at baseline. To establish the acute PH model, U46619 (0.9 μg/kg/min) was infused into the cephalic vein. After pretreatment with riociguat at a rate of 3 or 10 μg/kg/min for 10 min, U46619 was infused simultaneously for another 10 min. The drug sequences were randomized for each dog. A hemodynamic evaluation was performed 6 min after the beginning of each drug administration. The equilibrium time was established as 30 min between the drug infusions and before baseline measurement (Figure 1).

### 2.8. Statistical Analysis

Data were analyzed using GraphPad Prism software (GraphPad Software version 8.4.3). All data are shown as the mean ± SD. The results of the in vitro studies were statistically analyzed using an unpaired *t*-test. Differences between groups in in vivo studies were assessed using one-way repeated measures ANOVA (equal sample sizes between the groups) or linear mixed model estimated by the restricted maximum likelihood method (unequal sample sizes between the groups) followed by post hoc Tukey’s test. Statistical significance was set at *p* < 0.05.

## 3. Results

### 3.1. Effect of Riociguat on ET-1-Induced Contraction of Isolated Canine PA

**Figure 2:**
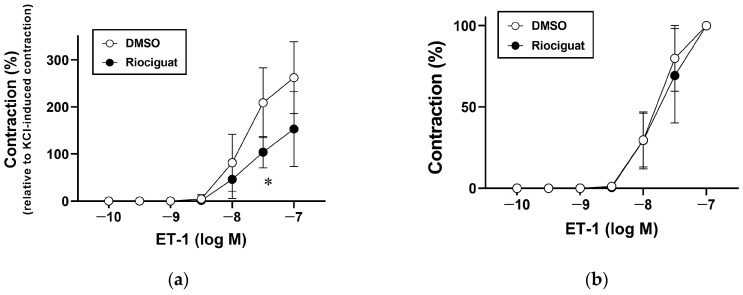
Effect of soluble guanylate cyclase stimulator riociguat on concentration–contraction relationship for endothelin (ET)-1 in an isolated endothelium-denuded canine pulmonary artery. Riociguat (100 nM, n = 5 rings) or dimethyl sulfoxide (DMSO; solvent of riociguat, n = 5 rings) was pretreated for 15 min before the application of ET-1. ET-1 (0.1 nM to 100 nM) was cumulatively applied. Each contraction was normalized to the (**a**) high-concentrated KCl- or (**b**) ET-1-induced maximal contraction. Results were expressed as means ± SD. * *p* < 0.05 vs. DMSO.

In isolated endothelium-denuded canine PA, the cumulative administration of ET-1 induced dose-dependent contractions (n = 4 rings; Figure 2). Pretreatment with riociguat significantly inhibited ET-1 (30 nM)-induced contractions [from 208.6 ± 74.6% (DMSO, solvent of riociguat, n = 4 rings) to 103.8 ± 33.4% (riociguat, n = 5 rings), *p* < 0.05; Figure 2a]. On the other hand, riociguat (pD_2_:7.7 ± 0.2, n = 5 rings; Figure 2b) had no effect on the contractile response to ET-1 (pD_2_:7.8 ± 0.2, n = 4 rings; Figure 2b). These results indicate that riociguat reduces the ET-1-induced contraction of canine PA without affecting the binding between ET-1 and its receptors.

### 3.2. Effect of Riociguat on cGMP Content in Isolated Canine PA

**Figure 3:**
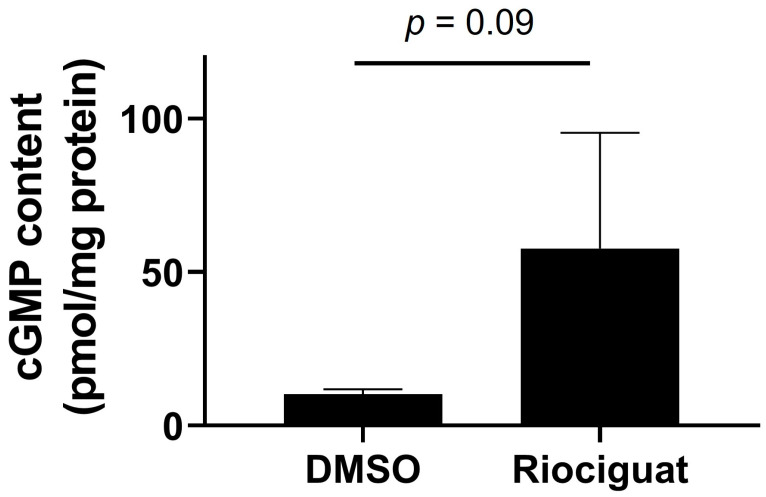
Effect of riociguat on production of cyclic guanosine monophosphate (cGMP) in an isolated endothelium-denuded canine pulmonary artery. After the vessels were treated with riociguat (100 nM, n = 4 rings) for 15 min, its total protein lysates were harvested. The cGMP levels were determined by ELISA. Result was expressed as means ± SD.

We further examined the effect of riociguat on cGMP levels in isolated endothelium-denuded canine PA. In contrast with the DMSO group (10.2 ± 1.6 pmol/mg protein, n = 3 rings; Figure 3), riociguat increased cGMP content in the PA (57.7 ± 37.7 pmol/mg protein, *p* = 0.09, n = 4 rings; Figure 3).

### 3.3. Effects of Riociguat on Elevated PAP in U46619-Induced Acute PH Model Dog

**Figure 4:**
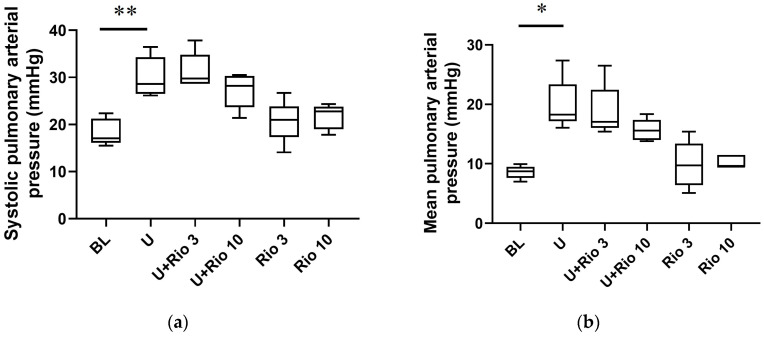

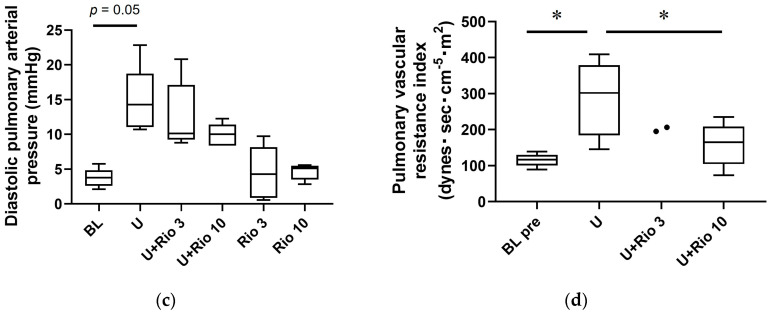
Effect of riociguat on pulmonary arterial pressure of dogs with U46619-induced pulmonary hypertension. A Swan–Ganz thermodilution catheter was inserted through the jugular vein and advanced into the pulmonary artery in anesthetized dogs. Thromboxane A2 analog U46619 (U; 0.9 μg/kg/min) was infused under simultaneous injection of riociguat (Rio; 3 or 10 μg/kg/min). Systolic (**a**) and diastolic (**c**) pulmonary arterial pressure (PAP) were measured, and mean PAP (**b**) and pulmonary vascular resistance (PVR) index (**d**) were calculated. In the box plots, the box indicates the interquartile range (25th to 75th percentiles), and the inner line indicates the median (50th percentile); the whiskers are located at the maximum and minimum observation. The PVR index of U46619 + riociguat infusion of 3 μg/kg per minute (U + Rio3) group (n = 2) was shown as dot plot. * *p* < 0.05, ** *p* < 0.01.

In contrast with the baseline [systolic pressure (sys):18.3 ± 2.8 mmHg, mean pressure (mean): 8.6 ± 1.1 mmHg, diastolic pressure (dia): 3.7 ± 1.3 mmHg, n = 5; Figure 4a–c and Table 1], U46619 increased the PAP (sys: 30.0 ± 4.3 mmHg, mean: 19.9 ± 4.4 mmHg, dia: 14.8 ± 4.8 mmHg, *p* < 0.01, n = 5; Figure 4a–c and Table 1). Riociguat (infusion rate: 10 μg/kg/min) inhibited it (sys: 27.2 ± 3.7 mmHg, mean: 15.7 ± 1.8 mmHg, dia: 9.9 ± 1.6 mmHg, n = 5; Figure 4a–c and Table 1). In addition, U46619 (285.6 ± 103.8 dynes·s·cm^−5^·m^2^, *p* < 0.05, n = 5; Figure 4d and Table 1) significantly increased the PVR index compared to baseline (115.8 ± 18.1 dynes·s·cm^−5^·m^2^, n = 5; Figure 4d and Table 1), which was significantly inhibited by riociguat (infusion rate:10 μg/kg/min; 158.3 ± 59.7 dynes·s·cm^−5^·m^2^, *p* < 0.05, n = 5; Figure 4d and Table 1).

### 3.4. Effects of Riociguat on SAP in U46619-Induced Acute PH Model Dog

In contrast with baseline (sys: 88.2 ± 10.0 mmHg, mean: 74.4 ± 10.8 mmHg, dia: 62.8 ± 9.7 mmHg, n = 5; Table 1), U46619 increased the SAP (sys: 101.8 ± 12.9 mmHg, mean: 86.2 ± 15.1 mmHg, dia: 74.8 ± 12.4 mmHg, n = 5; Table 1). Riociguat slightly inhibited it [(3 μg/kg/min; sys: 96.8 ± 8.1 mmHg, mean: 80.2 ± 8.1 mmHg, dia: 66.2 ± 8.4 mmHg, n = 5), (10 μg/kg/min; sys: 95.8 ± 8.0 mmHg, mean: 81.0 ± 8.7 mmHg, dia: 66.6 ± 8.4 mmHg, n = 5); Table 1]. In addition, U46619 (1559.7 ± 657.2 dynes·s·cm^−5^·m^2^, n = 5; Table 1) increased the SVR index compared to baseline (1228.2 ± 190.5 dynes·s·cm^−5^·m^2^, n = 5; Table 1), which was inhibited by riociguat [(3 μg/kg/min; 1054.0 ± 331.2 dynes·s·cm^−5^·m^2^, n = 5), (10 μg/kg/min; 1016.2 ± 294.5 dynes·s·cm^−5^·m^2^, n = 5); Table 1]. On the other hand, SAP was not affected by the administration of riociguat alone [(3 μg/kg/min; sys: 90.4 ± 12.0 mmHg, mean: 76.2 ± 13.4 mmHg, dia: 61.2 ± 12.3 mmHg, n = 5), (10 μg/kg/min; sys: 90.6 ± 10.6 mmHg, mean: 75.2 ± 11.7 mmHg, dia: 61.6 ± 11.1 mmHg, n = 5); Table 1].

### 3.5. Effects of Riociguat on CO in U46619-Induced Acute PH Model Dog

In contrast with baseline (2.4 ± 0.6 L/min, n = 5; Table 1), U46619 had no effect on CO (2.3 ± 0.9 L/min, n = 5; Table 1). Riociguat (infusion rate:10 μg/kg/min) increased CO under U46619 administration (3.5 ± 1.8 L/min, n = 5; Table 1).

## 4. Discussion

The sGC stimulator riociguat is a pulmonary vasodilator used to treat pulmonary arterial hypertension and chronic thromboembolic PH in humans. However, basic and clinical studies on the efficacy of riociguat against PH have not been performed in veterinary medicine. Thus, in this study, we investigated the effects of riociguat on the contractility of isolated canine PA and hemodynamics of PH model dogs.

In endothelium-denuded canine PA, riociguat significantly inhibited ET-1 (30 nM)-induced contractions without affecting the contractile response to ET-1 (Figure 2). ET-1 is a vasoconstrictor of PA, which promotes the pathogenesis of PH. It has been reported that in dogs with PH, serum pro-ET-1 levels [23] and ET-1 expression in the lung tissue [24] increased compared to those in the control group. In addition, the cGMP content in isolated canine PA was increased by the riociguat treatment (Figure 3). sGC stimulators, including riociguat, induce the endothelium-independent relaxation of PA smooth muscle cells via direct activation of sGC and subsequent elevation of intracellular cGMP concentration [17,18]. An sGC inhibitor, 1H-[1,2,4]oxadiazolo[4,3-1]-quinoxalin-1-one attenuated NO donor-induced relaxation of isolated canine PA through the inhibition of cGMP generation [25]. These results demonstrated that riociguat has an endothelium-independent relaxant effect on canine PA via an increase in cGMP content. In the pathologic condition of PH with vascular endothelial dysfunction, the cGMP content of PA is decreased due to the depletion of endothelium-derived NO [14,26], which can lead to diminishing the vasorelaxant effect of PDE5 inhibitors. Therefore, riociguat may have clinical efficacy against dogs with refractory PH that is resistant to PDE5 inhibitors.

We also examined the effects of riociguat on the hemodynamics of dogs with U46619-induced PH. An acute and transient PH animal model was established by injection of U46619, a thromboxane A2 analog [19,20]. Thromboxane A2 is an endogenous contracting factor for PA. U46619 induced the elevation of the PVR index and PAP in dogs, which was inhibited by prior infusion of riociguat (Figure 4 and Table 1). However, riociguat inadequately inhibited U46619-induced elevation of systolic PAP, regardless of the decreased PVR index. This may be due to a riociguat-induced increase in CO, as will be described later. In fact, riociguat inhibited the diastolic PAP elevation induced by the U46619 injection. Riociguat has been reported to decrease PVR and PAP in clinical examinations of patients with PH [27]. In addition, it has been shown that riociguat inhibited right ventricular fibrosis and dysfunction in mice with PA constriction-induced right heart failure [28], which demonstrates that riociguat has inhibitory effects on the development of PH. Although riociguat slightly diminished the U46619-induced elevation of SAP, it had no effect on the basal SAP (Table 1). The U46619-induced increase in the SVR index was decreased by riociguat injection, whereas CO was increased (Table 1). Because SAP is equal to the product of SVR and CO [29], increased CO caused by riociguat may cancel out the decrease in basal SAP associated with decreasing SVR. In human PH patients, riociguat increased right ventricular contraction, measured by echocardiography, without affecting the mean PAP [27], indicating that the increased cardiac contraction was caused by the direct effect of riociguat, but not secondary to a decrease in the pressure road. Thus, it is possible that riociguat directly increased CO. The present results suggest that riociguat can be used as a pulmonary vasodilator with almost no effect on basal SAP in dogs.

In an in vitro study using isolated canine PA, 100 nM riociguat inhibited the ET-1-induced increase in PA contraction. Since the normal circulating blood volume of dogs is estimated to be 85 mL/kg [30], the concentration of riociguat was converted to approximately 3.6 μg/kg in dogs. Therefore, the infusion rate of riociguat was determined to be 3–10 μg/kg/min in vivo.

This study has several limitations. Firstly, we only investigated the acute effects of riociguat on hemodynamics in dogs. Secondly, combined administration of riociguat and other vasodilators was not examined. In fact, in veterinary clinical medicine, the patients with PH have already taken some vasodilators. In human medicine, a concomitant use of riociguat with other vasodilators including PDE5 inhibitor and NO donor was reported to induce significant hypotension [31]. Then, further study to investigate the chronic effect and/or combined use of riociguat is warranted in dogs.

## 5. Conclusions

The present study revealed for the first time that riociguat inhibited the ET-1-induced contraction of isolated endothelium-denuded canine PA, possibly by increasing intracellular cGMP. Moreover, riociguat attenuated the elevation of PAP without decreasing the basal SAP in U46619-induced acute PH model dogs. These results suggest that riociguat could be used as a novel pulmonary vasodilator without lowering SAP in veterinary clinical medicine.

## Figures and Tables

**Table 1:** Effects of riociguat on the hemodynamics of dogs with U46619-induced pulmonary hypertension. CO: cardiac output; CVP: central venous pressure; d: diastolic; HR: heart rate; m: mean; PAP: pulmonary arterial pressure; PAWP: pulmonary arterial wedge pressure; PVR: pulmonary vascular resistance; Rio 3: riociguat infusion of 3 μg/kg per minute; Rio 10: riociguat infusion of 10 μg/kg per minute; SAP: systemic arterial pressure; SVR: systemic vascular resistance; s: systolic; U: U46619 alone; U + Rio 3: U46619 + riociguat infusion of 3 μg/kg per minute; U + Rio 10: U46619 + riociguat infusion of 10 μg/kg per minute. The data are shown as mean ± SD. * *p* < 0.05 vs. baseline, ** *p* < 0.01 vs. baseline, # *p* < 0.05 vs. U.

	Baseline	U	U + Rio 3	U + Rio 10	Rio 3	Rio 10
HR (bpm)	114.0 ± 11.8 (n = 5)	119.0 ± 18.2 (n = 5)	132.8 ± 24.8 (n = 5)	138.0 ± 19.2 (n = 5)	139.2 ± 30.5 (n = 5)	134.0 ± 23.8 (n = 5)
sPAP (mmHg)	18.3 ± 2.8 (n = 5)	30.0 ± 4.3 ** (n = 5)	31.3 ± 3.8 (n = 5)	27.2 ± 3.7 (n = 5)	20.7 ± 4.5 (n = 5)	21.7 ± 2.6 (n = 5)
mPAP (mmHg)	8.6 ± 1.1 (n = 5)	19.9 ± 4.4 * (n = 5)	18.8 ± 4.4 (n = 5)	15.7 ± 1.8 (n = 5)	9.9 ± 3.9 (n = 5)	10.3 ± 1.1 (n = 5)
dPAP (mmHg)	3.7 ± 1.3 (n = 5)	14.8 ± 4.8 (n = 5)	12.6 ± 4.9 (n = 5)	9.9 ± 1.6 (n = 5)	4.5 ± 3.8 (n = 5)	4.6 ± 1.1 (n = 5)
sSAP (mmHg)	88.2 ± 10.0 (n = 5)	101.8 ± 12.9 * (n = 5)	96.8 ± 8.1 (n = 5)	95.8 ± 8.0 (n = 5)	90.4 ± 12.0 (n = 5)	90.6 ± 10.6 (n = 5)
mSAP (mmHg)	74.4 ± 10.8 (n = 5)	86.2 ± 15.1 (n = 5)	80.2 ± 8.1 (n = 5)	81.0 ± 8.7 (n = 5)	76.2 ± 13.4 (n = 5)	75.2 ± 11.7 (n = 5)
dSAP (mmHg)	62.8 ± 9.7 (n = 5)	74.8 ± 12.4 (n = 5)	66.2 ± 8.4 (n = 5)	66.6 ± 8.4 (n = 5)	61.2 ± 12.3 (n = 5)	61.6 ± 11.1 (n = 5)
mPAWP (mmHg)	1.6 ± 0.8 (n = 5)	4.2 ± 1.7 (n = 5)	3.5 ± 2.8 (n = 4)	3.5 ± 1.1 (n = 5)	1.4 ± 1.5 (n = 5)	2.1 ± 1.5 (n = 5)
mCVP (mmHg)	0.5 ± 1.0 (n = 5)	2.8 ± 1.9 (n = 5)	3.0 ± 2.0 (n = 5)	1.3 ± 1.0 (n = 5)	−0.6 ± 2.4 (n = 5)	0.2 ± 1.4 (n = 5)
CO (L/min)	2.4 ± 0.6 (n = 5)	2.3 ± 0.9 (n = 5)	2.9 ± 0.7 (n = 3)	3.5 ± 1.8 (n = 5)	-	-
PVR index (dynes·s·cm^−5^·m^2^)	115.8 ± 18.1 (n = 5)	285.6 ± 103.8 * (n = 5)	200.9 ± 7.8 (n = 2)	158.3 ± 59.7 # (n = 5)	-	-
SVR index (dynes·s·cm^−5^·m^2^)	1228.2 ± 190.5 (n = 5)	1559.7 ± 657.2 (n = 5)	1054.0 ± 331.2 (n = 3)	1016.2 ± 294.5 (n = 5)	-	-

## Data Availability

Detailed data of the present study are available from the corresponding author upon reasonable request.

## References

[1] Deng J. (2021). Clinical Application of Pulmonary Vascular Resistance in Patients with Pulmonary Arterial Hypertension. J. Cardiothorac. Surg..

[2] Al-Omary M.S., Sugito S., Boyle A.J., Sverdlov A.L., Collins N.J. (2020). Pulmonary Hypertension Due to Left Heart Disease: Diagnosis, Pathophysiology, and Therapy. Hypertension.

[3] Rosenzweig E.B., Krishnan U. (2021). Congenital Heart Disease-Associated Pulmonary Hypertension. Clin. Chest Med..

[4] Reinero C., Visser L.C., Kellihan H.B., Masseau I., Rozanski E., Clercx C., Williams K., Abbott J., Borgarelli M., Scansen B.A. (2020). ACVIM Consensus Statement Guidelines for the Diagnosis, Classification, Treatment, and Monitoring of Pulmonary Hypertension in Dogs. J. Vet. Intern. Med..

[5] Glaus T.M., Soldati G., Maurer R., Ehrensperger F. (2004). Clinical and Pathological Characterisation of Primary Pulmonary Hypertension in a Dog. Vet. Rec..

[6] Rawlings C.A., Farrell R.L., Mahood R.M. (1990). Morphologic Changes in the Lungs of Cats Experimentally Infected with Dirofilaria Immitis. Response to Aspirin. J. Vet. Intern. Med..

[7] Ray L., Mathieu M., Jespers P., Hadad I., Mahmoudabady M., Pensis A., Motte S., Peters I.R., Naeije R., McEntee K. (2008). Early Increase in Pulmonary Vascular Reactivity with Overexpression of Endothelin-1 and Vascular Endothelial Growth Factor in Canine Experimental Heart Failure. Exp. Physiol..

[8] Pulido T., Zayas N., de Mendieta M.A., Plascencia K., Escobar J. (2016). Medical Therapies for Pulmonary Arterial Hypertension. Heart Fail. Rev..

[9] Zhao Y., Vanhoutte P.M., Leung S.W.S. (2015). Vascular Nitric Oxide: Beyond ENOS. J. Pharmacol. Sci..

[10] Cohen A.H., Hanson K., Morris K., Fouty B., McMurtry I.F., Clarke W., Rodman D.M. (1996). Inhibition of Cyclic 3′-5′-Guanosine Monophosphate-Specific Phosphodiesterase Selectively Vasodilates the Pulmonary Circulation in Chronically Hypoxic Rats. J. Clin. Investig..

[11] Yamamoto T., Wada A., Tsutamoto T., Ohnishi M., Horie M. (2004). Long-Term Treatment with a Phosphodiesterase Type 5 Inhibitor Improves Pulmonary Hypertension Secondary to Heart Failure through Enhancing the Natriuretic Peptides-CGMP Pathway. J. Cardiovasc. Pharmacol..

[12] Fullerton D.A., Mitchell M.B., Jones D.N., Maki A., McIntyre R.C. (1996). Pulmonary Vasomotor Dysfunction Is Produced with Chronically High Pulmonary Blood Flow. J. Thorac. Cardiovasc. Surg..

[13] Kurakula K., Smolders V.F.E.D., Tura-Ceide O., Wouter Jukema J., Quax P.H.A., Goumans M.J. (2021). Endothelial Dysfunction in Pulmonary Hypertension: Cause or Consequence?. Biomedicines.

[14] Giaid A., Saleh D. (1995). Reduced Expression of Endothelial Nitric Oxide Synthase in the Lungs of Patients with Pulmonary Hypertension. N. Engl. J. Med..

[15] Johnson L.R., Stern J.A. (2020). Clinical Features and Outcome in 25 Dogs with Respiratory-Associated Pulmonary Hypertension Treated with Sildenafil. J. Vet. Intern. Med..

[16] Kellum H.B., Stepien R.L. (2007). Sildenafil Citrate Therapy in 22 Dogs with Pulmonary Hypertension. J. Vet. Intern. Med..

[17] Schermuly R.T., Stasch J.P., Pullamsetti S.S., Middendorff R., Müller D., Schlüter K.D., Dingendorf A., Hackemack S., Kolosionek E., Kaulen C. (2008). Expression and Function of Soluble Guanylate Cyclase in Pulmonary Arterial Hypertension. Eur. Respir. J..

[18] Næsheim T., How O.J., Myrmel T. (2021). Hemodynamic Effects of a Soluble Guanylate Cyclase Stimulator, Riociguat, and an Activator, Cinaciguat, During NO-Modulation in Healthy Pigs. J. Cardiovasc. Pharmacol. Ther..

[19] Gong F., Shiraishi H., Kikuchi Y., Hoshina M., Ichihashi K., Sato Y., Momoi M.Y. (2000). Inhalation of Nebulized Nitroglycerin in Dogs with Experimental Pulmonary Hypertension Induced by U46619. Pediatr. Int..

[20] Hori Y., Kondo C., Matsui M., Yamagishi M., Okano S., Chikazawa S., Kanai K., Hoshi F., Itoh N. (2014). Effect of the Phosphodiesterase Type 5 Inhibitor Tadalafil on Pulmonary Hemodynamics in a Canine Model of Pulmonary Hypertension. Vet. J..

[21] Freitas C.F., Morganti R.P., Annichino-Bizzacchi J.M., De Nucci G., Antunes E. (2007). Effect of BAY 41-2272 in the Pulmonary Hypertension Induced by Heparin-Protamine Complex in Anaesthetized Dogs. Clin. Exp. Pharmacol. Physiol..

[22] Ricco Pereira C. (2020). Body Surface Area Calculation for Dogs and Cats Using LiDCO and PICCO Monitors. J. Vet. Emerg. Crit. Care.

[23] Fukumoto S., Hanazono K., Miyasho T., Endo Y., Kadosawa T., Iwano H., Uchide T. (2014). Serum Big Endothelin-1 as a Clinical Marker for Cardiopulmonary and Neoplastic Diseases in Dogs. Life Sci..

[24] Qingyan Z., Xuejun J., Yanhong T., Zixuan D., Xiaozhan W., Xule W., Zongwen G., Wei H., Shengbo Y., Congxin H. (2015). Beneficial Effects of Renal Denervation on Pulmonary Vascular Remodeling in Experimental Pulmonary Artery Hypertension. Rev. Esp. Cardiol. (Engl. Ed.).

[25] Kwak Y.L., Jones K.A., Warner D.O., Perkins W.J. (2006). Prolonged Relaxation Consistent with Persistent Soluble Guanylyl Cyclase Activation in Canine Pulmonary Artery Following Brief Treatment with Nitric Oxide Donors. Life Sci..

[26] Morita K., Ogawa Y., Tobise K. (1996). Effect of Endothelium of Pulmonary Artery Vasoreactivity in Monocrotaline-Induced Pulmonary Hypertensive Rats. Jpn. Circ. J..

[27] Murata M., Kawakami T., Kataoka M., Moriyama H., Hiraide T., Kimura M., Endo J., Kohno T., Itabashi Y., Fukuda K. (2021). Clinical Significance of Guanylate Cyclase Stimulator, Riociguat, on Right Ventricular Functional Improvement in Patients with Pulmonary Hypertension. Cardiology.

[28] Rai N., Veeroju S., Schymura Y., Janssen W., Wietelmann A., Kojonazarov B., Weissmann N., Stasch J.P., Ghofrani H.A., Seeger W. (2018). Effect of Riociguat and Sildenafil on Right Heart Remodeling and Function in Pressure Overload Induced Model of Pulmonary Arterial Banding. Biomed. Res. Int..

[29] Magder S. (2018). The Meaning of Blood Pressure. Crit. Care.

[30] Jahr J.S., Lurie F., Bezdikian V., Driessen B., Gunther R.A. (2008). Measuring Circulating Blood Volume Using Infused Hemoglobin-Based Oxygen Carrier (Oxyglobin) as an Indicator: Verification in a Canine Hypovolemia Model. Am. J. Ther..

[31] Frey R., Becker C., Saleh S., Unger S., van der Mey D., Mück W. (2018). Clinical Pharmacokinetic and Pharmacodynamic Profile of Riociguat. Clin. Pharmacokinet..

